# An effective hyper-parameter can increase the prediction accuracy in a single-step genetic evaluation

**DOI:** 10.3389/fgene.2023.1104906

**Published:** 2023-06-08

**Authors:** Mehdi Neshat, Soohyun Lee, Md. Moksedul Momin, Buu Truong, Julius H. J. van der Werf, S. Hong Lee

**Affiliations:** ^1^ Australian Centre for Precision Health, University of South Australia, Adelaide, SA, Australia; ^2^ UniSA Allied Health and Human Performance, University of South Australia, Adelaide, SA, Australia; ^3^ South Australian Health and Medical Research Institute (SAHMRI), Adelaide, SA, Australia; ^4^ Division of Animal Breeding and Genetics, National Institute of Animal Science (NIAS), Cheonan, Republic of Korea; ^5^ Department of Genetics and Animal Breeding, Faculty of Veterinary Medicine, Chattogram Veterinary and Animal Sciences University (CVASU), Chattogram, Bangladesh; ^6^ Cardiovascular Research Centre, Massachusetts General Hospital, Boston, MA, United States; ^7^ Center for Genomic Medicine, Massachusetts General Hospital, Boston, MA, United States; ^8^ Program in Medical and Population Genetics and the Cardiovascular Disease Initiative, Broad, Institute of Harvard and Massachusetts Institute of Technology (MIT), Cambridge, MA, United States; ^9^ School of Environmental and Rural Science, University of New England, Armidale, NSW, Australia

**Keywords:** genomic prediction, single-step genetic evaluation, hyper-parameters, scale factor, harmonised matrix

## Abstract

The H-matrix best linear unbiased prediction (HBLUP) method has been widely used in livestock breeding programs. It can integrate all information, including pedigree, genotypes, and phenotypes on both genotyped and non-genotyped individuals into one single evaluation that can provide reliable predictions of breeding values. The existing HBLUP method requires hyper-parameters that should be adequately optimised as otherwise the genomic prediction accuracy may decrease. In this study, we assess the performance of HBLUP using various hyper-parameters such as blending, tuning, and scale factor in simulated and real data on Hanwoo cattle. In both simulated and cattle data, we show that blending is not necessary, indicating that the prediction accuracy decreases when using a blending hyper-parameter <1. The tuning process (adjusting genomic relationships accounting for base allele frequencies) improves prediction accuracy in the simulated data, confirming previous studies, although the improvement is not statistically significant in the Hanwoo cattle data. We also demonstrate that a scale factor, α , which determines the relationship between allele frequency and per-allele effect size, can improve the HBLUP accuracy in both simulated and real data. Our findings suggest that an optimal scale factor should be considered to increase prediction accuracy, in addition to blending and tuning processes, when using HBLUP.

## 1 Introduction

Genomic prediction can achieve a more accurate prediction of additive genetic values at an early life stage, compared to the conventional pedigree-based prediction. Genomic prediction has been applied to a broad range of disciplines, including animal breeding (Hayes et al., 2009) and human disease risk prediction (Abraham et al., 2016; Inouye et al., 2018; Khera et al., 2018). The accuracy of genomic prediction is important, which depends on several factors such as marker density, linkage disequilibrium (LD) between the quantitative trait loci (QTLs) and markers, the sample size of reference, the heritability of the trait, the number of QTLs, and the distribution of QTL effects. The prediction accuracy is also determined by the method used (Yan et al., 2022).

Genomic prediction requires genotypic information for both discovery and target samples. Genome-wide single nucleotide polymorphisms (SNPs) are typically used to estimate the genomic relationship matrix (GRM) for the genotyped samples so that breeding values (in livestock) can be estimated for the target samples, given the phenotypic information of discovery samples (De Los Campos et al., 2009; VanRaden et al., 2009). In many cases, we may have individuals with useful phenotypic information that are not genotyped, but they may be linked with genotyped samples through a pedigree, i.e., missing genotype data. To address this problem, a single-step genomic best linear unbiased prediction (ssGBLUP) method was introduced, in which phenotypic information on both genotyped and non-genotyped individuals in the pedigree can be used simultaneously to maximise the prediction accuracy of genotyped target individuals (Legarra et al., 2009; Christensen and Lund, 2010; Christensen et al., 2012; McWhorter et al., 2022).

SsGBLUP uses an H-matrix that is a harmonised matrix of a pedigree-based numerator relationship matrix (NRM) and a GRM; therefore, we will use the term H-matrix best linear unbiased prediction (HBLUP). The H-matrix allows us to use the information of non-genotyped individuals in genomic prediction using a data augmentation technique (see (Legarra et al., 2009; Misztal et al., 2009) and Legarra et al., 2014). HBLUP has been widely used in the genetic evaluation of livestock and has been employed in the national genetic evaluation program in many countries (Gao et al., 2012; McMillan and Swan, 2017; Brown et al., 2018; Chung et al., 2018; Johnston et al., 2018; Meyer et al., 2018; Teissier et al., 2018; Oliveira et al., 2019; Mäntysaari et al., 2020; Alkhoder and Liu, 2021). There are numerous studies reporting that HBLUP outperforms traditional GBLUP (Baloche et al., 2014; Gao et al., 2018; Gowane et al., 2019; Mancisidor et al., 2021).

In HBLUP, there are two main hyper-parameters that can determine its performance. First, blending is one of the hyper-parameters that can provide a weighted sum of genomic and numerator relationships, using an arbitrary weight typically ranging from 0.5 to 0.99 (Meyer et al., 2018). This process is essential because it ensures GRM, which is a positive definite matrix, avoids numerical problems in HBLUP (VanRaden, 2008; Legarra et al., 2009). Second, tuning is another important hyper-parameter that can adjust GRM, accounting for the allele frequencies in the base population that are inferred from the information of NRM (Legarra et al., 2009; Misztal et al., 2009; Chen et al., 2011; Vitezica et al., 2011). Note that GRM is typically based on genotyped samples in the last few generations, whereas NRM includes the information of founders in the base population through the pedigree. Third, a scale factor is a novel hyper-parameter for HBLUP to be introduced in this study, which can generate different kinds of GRMs, accounting for the relationship between allele frequency and per-allele effect size, i.e., per-allele effect sizes vary, depending on a function proportional to [*p* (1 − *p*)] ^*α*^, where *p* is the allele frequency (Speed et al., 2012; Speed et al., 2017; Schoech et al., 2019; Momin et al., 2023). Negative α values indicate larger effect sizes for rare variants, and the choice of α may determine the HBLUP accuracy, i.e., an optimal α can increase the accuracy. Bouwman et al. (2017) considered alternative scale factors in GBLUP, which were applied to a dairy cattle dataset. However, they did not test the impact of alternative scale factors in HBLUP; therefore, it is unclear how the scale factor, as a hyper-parameter, determines the HBLUP accuracy.

In this study, we investigate the three hyper-parameters, blending, tuning, and α , to assess how they affect HBLUP accuracy, using simulated and real data. There are several tuning methods (Legarra et al., 2009; Chen et al., 2011; Vitezica et al., 2011; Meyer et al., 2018) among which we test two most frequently used approaches, i.e., methods by Chen et al. (2011) (Chen et al., 2011) and Vitezica et al. (2011) (Vitezica et al., 2011), referred to as tune = 1 and 2 in this study. For blending, we investigate a wide range of weighting factors ( θ ) to assess the performance of HBLUP. In the analyses, we use the direct Average Information algorithm (Lee and Van Der Werf, 2006; Yang et al., 2011) that is robust to the numerical problem caused by non-positive definite GRM so that we can assess all kinds of weighting factors in blending, including θ = 1. We also assess HBLUP performance, varying the scale factor, ranging from α = −1.5 to 1.5, in the estimation of GRM. We consider the three hyper-parameters simultaneously to obtain optimal values for blending, tuning, and α , using a grid search method (Bergstra et al., 2011). Then, the performance of HBLUP with optimal values is compared to performances with less optimal values.

## 2 Material and methods

### 2.1 Simulated data

QMSim software (Sargolzaei and Schenkel, 2009) was used for simulation since it can efficiently generate a large-scale dataset including genotypic and pedigree information. We simulated three different scenarios that differed in terms of the effective population size, mating design, and family structure. Two different effective population sizes are determined at 100 and 1,000 individuals with 100 generations in order to mimic livestock (a half-sib design) and human (a full-sib design) populations.

I. The historical population consists of 100 generations. For the initial 95 generations, the effective population size ( $N_{e}$ ) keeps fixed at 100 individuals, consisting of 50 female individuals and 50 male individuals. Two offspring are generated with random selection and random mating of parents. In the following five generations (95th–100th), the number of progenies gradually increased to 1,000. In the last generation of the historical population (the 100th generation), we randomly select 50 male individuals and 500 female individuals as the founders, and each male individual is mated with ten female individuals and each female individual produced two offspring (i.e., a half-sib design). The current population consists of five generations with 1,000 offspring in each generation (101–105th generations), which is used for the main analyses. The details of applied parameters in the simulation of genotypic and pedigree data are listed in Table 1. The steps to simulate the historical and current populations are illustrated in Supplementary Figure S1.
II. In the second simulation scenario, $N_{e}$ = 1,000 is used (500 female individuals and 500 male individuals) with a historical population of 100 generations. The population size for each generation in the historical population with 100 generations is constant (N = 1,000). In the subsequent five generations (101st–105th), each male individual is mated with one female individual and each female individual produced two offspring (i.e., a full-sib design), and 1,000 offspring were generated in total. Thus, the founder population size is 1,000.
III. In the third scenario, $N_{e}$ and the number of generations in the historical population are the same as in the first scenario ( $N_{e}$ = 100 with 100 generations). However, In the last generation of the historical population (100th) and the subsequent five generations (101st–105th), the mating design and family structure are the same as the second scenario, i.e., one male individual is mated with one female individual to produce two progeny per mating (full-sib design), producing 1,000 offspring in total in each generation.

**TABLE 1:** Parameters of historical population and genotyping data simulation in the first scenario using QMSim software.

QMSim parameters	Value
Litter size	2
The proportion of male progeny	0.5
Mating design	random (*rnd*)
Selection design	random (*rnd*)
Number of SNPs	$9\times 10^{3}$
Number of Chromosomes	30
Chromosome length (cM)	100
Number of marker loci on the chromosome	300
Marker positions	random (*rnd*)
Marker allele frequencies	*equal*
Marker mutation rate	$2.8\times 10^{-8}$

In order to simulate the phenotypes of a complex trait based on the simulated genotyped data, we used a model,

$$y_{i}=Z_{i}u+e_{i}$$ (1)

where $y_{i}$ is the phenotypic value, $Z_{i}$ is the vector of SNP genotypes and $e_{i}$ is the residual effect for the $i^{th}$ individual, and **u** is the vector of SNP effects. In this phenotypic simulation, we randomly selected 1,000 SNPs as causal variants, and **u** was drawn from a normal distribution such that the mean and variance of the genetic effects are $mean\left(Z_{i}u\right)=0$ and $var\left(Z_{i}u\right)=h^{2}$ . The residual effects were generated from a normal distribution with mean = 0 and variance $=1-h^{2}$ . In the phenotypic simulation, the SNP effects, **u**, are scaled by [2*p* (1 − *p*)]^*α*^, considering a non-negligible relationship between allele frequency and per-allele effect size (Speed et al., 2012; Speed et al., 2017; Schoech et al., 2019; Momin et al., 2023), which is a function of alpha ranging from −1.5 to 1.5 in the simulation.

In the HBLUP analysis, for three simulation scenarios, it is assumed that the pedigree information is available for the last five generations (101–105th generations), and the genotypic information is available for the individuals from the last two generations (104–105th generations), noting that the sample size in each of the last five generations is 1,000. Furthermore, it is noted that the phenotypes are available for all individuals. We conducted 3,000 replicates of the simulations under three different scenarios with specified simulation parameters. By running multiple replicates, we were able to estimate the variance and uncertainty in the results and obtain a more accurate assessment of the effects of different factors on the population. Replicating the simulation multiple times is a common practice in simulation studies as it can increase the reliability and validity of the results by reducing the impact of chance events and providing a more robust assessment of the effects of the factors being studied.

### 2.2 Real data

#### 2.2.1 Hanwoo cattle data

In this study, we applied statistical analyses to genotypic and phenotypic data from Hanwoo beef cattle. The total number of animals with pedigree information was 84,020, and among them, 13,800 animals were genotyped for 52,791 genome-wide SNPs, and 25,502 animals were recorded for their phenotypes. The number of animals available for both genotypic and phenotypic information was 9,072. The following criteria were applied for quality control (QC) using PLINK: minor allele frequency below 0.01 (MAF), filtering SNPs with a call rate lower than 95% (GENO = 0.05), individual missingness more than 5% (MIND = 0.05), and Hardy–Weinberg Equilibrium *p*-value threshold lower than 1e-04 (HWE). After QC, the number of individuals did not change, and the SNPs number was 42,795. The Hanwoo beef cattle data included five carcass traits: carcass weight, eye muscle area, back fat thickness, marbling score, and adjusted 12 months weight. The total number of animals with non-missing records for each carcass trait with and without genotypic information can be seen in Table 2.

**TABLE 2:** The number of individuals available for phenotypes with and without genotypic information for five carcass traits in the Hanwoo cattle dataset.

#	Traits	Phenotypic records	With genotype	Without genotype
1	Carcass weight (c_awgt in *Kg*)	7,833	4,607	3,226
2	Eye muscle area (c_ema in *cm* ^*2*^)	7,829	4,607	3,222
3	Back fat (c_bf in *mm*)	7,834	4,607	3,227
4	Marbling score (c_ms in *1–9*)	5,998	4,607	1,391
5	Adjusted 12 months’ weight (adj-w12)	18,654	9,072	9,582

**FIGURE 1:**
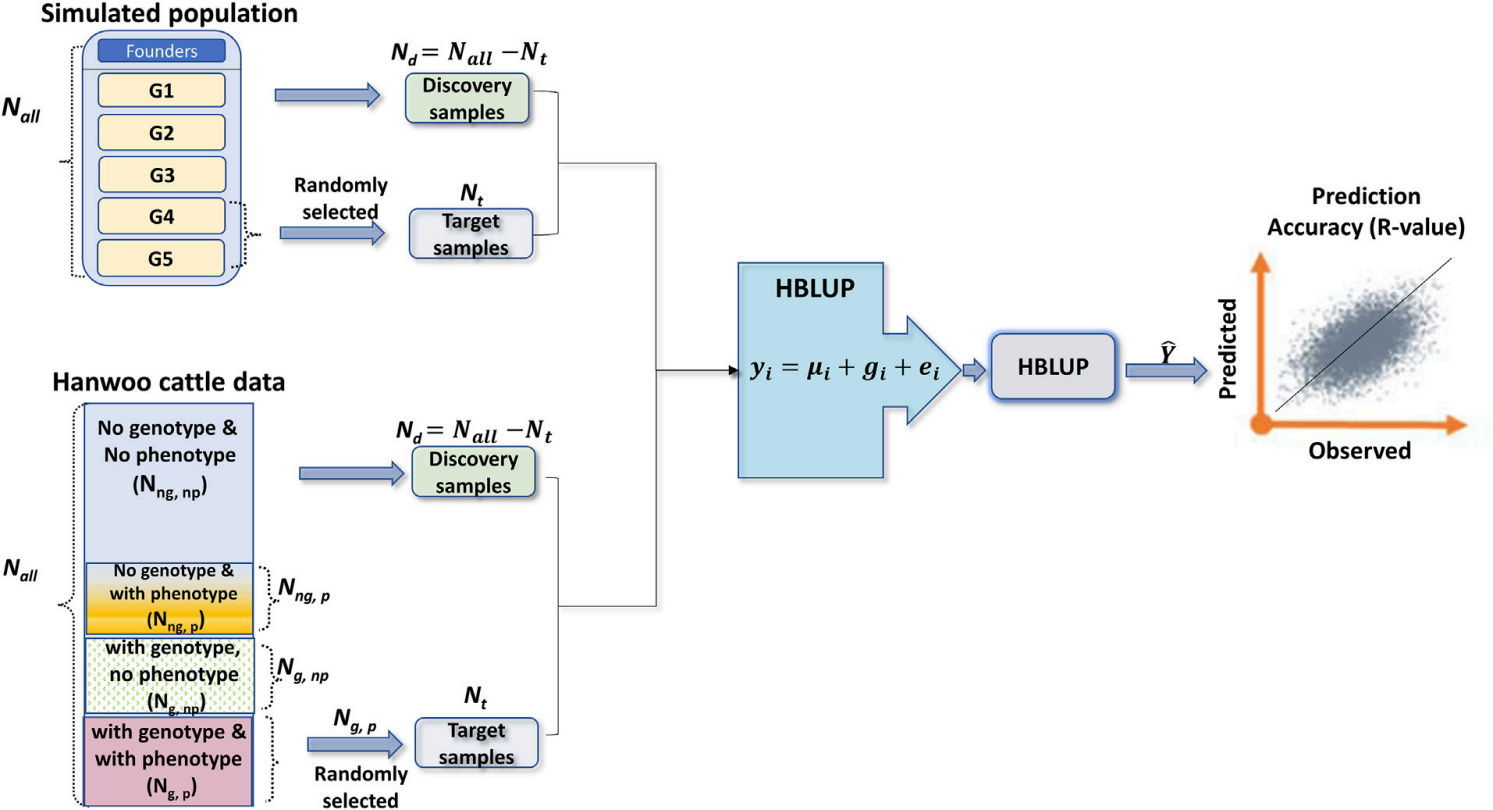
A diagram showing the experimental designs and how to select the target and discovery samples for simulated and Hanwoo cattle datasets. In the simulated dataset, the number of founders depends on the simulation scenarios ( $f_{n}$ = 550, 1,000, and 550 for simulation scenarios 1, 2, and 3). The sample size in each generation ( $G_{i}$ ) is 1,000. Therefore, the sample size in the whole population is $N_{all}$ = $\sum_{i=1}^{N}G_{i}+f_{n}$ . The sample sizes of target and discovery samples are denoted as $N_{t}$ and $N_{d}$ . In Hanwoo cattle data, the phenotypic and genotypic information is partly missing. The number of animals without genotype and phenotype ( $N_{ng,np}$ ), animals without genotype but with phenotype ( $N_{ng,p}$ ), animals with genotype but without phenotype ( $N_{g,np}$ ), and animals with both genotype and phenotype ( $N_{g,p}$ ) are shown in the diagram. $N_{g}$ is the total number of genotyped animals. In HBLUP, for the animals with both genotype and phenotype ( $N_{g,p}$ ), 5-fold cross-validation is applied, and each fold is selected as the target dataset ( $N_{t}$ ), and the remaining animals with phenotypes are used as the discovery samples ( $N_{d}$ ). The best linear unbiased predictions for the phenotypes of the target samples are obtained. In order to calculate the prediction accuracy, we used Pearson’s correlation coefficients between the true and predicted phenotypes for the target samples. It is noted that the target dataset is selected from the last generations (offspring) and should be predicted by the previous generations (discovery population).

In the HBLUP analysis for the Hanwoo cattle data, animals available for phenotypes and genotypes ( $N_{g,p}$ ) (see Table 2) are randomly divided into five groups. In a 5-fold cross-validation, one of the five groups is selected as the target dataset, and the remaining groups are used as the discovery dataset, which is repeated five times, and the averaged phenotypic prediction accuracy is calculated. The technical details of the training and validation of HBLUP can be seen in Figure 1.

### 2.3 Estimating NRM, GRM, and HRM

#### 2.3.1 Numerator relationship matrix

NRM is denoted as **A** which is estimated based on the pedigree and has been used in Henderson’s mixed model equation (Henderson, 1975) to obtain estimated breeding values. Following Legarra et al., 2014, **A** matrix can be formulated as follows.

$$A=\left[\begin{array}{cc} A_{11} & A_{12}\\ A_{21} & A_{22} \end{array}\right]$$ (2)

Where $A_{11}$ and $A_{22}$ denote the numerator relationships for the groups of non-genotyped and genotyped individuals, and $A_{12}$ and $A_{21}$ are the numerator relationships between non-genotyped and genotyped individuals.

#### 2.3.2 Scale factor ( α ) and GRM

Following Momin et al., 2023, the variance of the $i^{th}$ genetic variant (*v*_i_) can be expressed as a function of the allele substation effect ( u ) and the allele frequency ( $p_{i}$ ), which can be written as

$$Var\left(v_{i}\right)=2p_{i}\left(1-p_{i}\right)\gamma_{i}^{2}={\left[2p_{i}\left(1-p_{i}\right)\right]}^{1+2\alpha }\times {u_{i}}^{2}$$ (3)

where $\gamma_{i}=u_{i}\times {\left[2p_{i}\left(1-p_{i}\right)\right]}^{\alpha }$ is the allele effect size ( $u_{i}$ ) that can vary, depending on the allele frequency and the scale factor, α (Speed et al., 2012; Speed et al., 2017), which can be explained by evolutionary forces such as selections, mutations, immigrations, and genetic drift. In the classical model [36], α is assumed to be zero for all traits. Another widely used α value is α = −0.5, assuming that the genetic variance of the causal variant has a uniform distribution across the minor allele frequency spectrum. However, there have been reports that optimal α values vary, depending on traits and populations (Speed et al., 2012; Speed et al., 2017; Momin et al., 2023). Following Speed et al., 2020, the genomic relationship matrix can be formulated as a function of α , which can be written as

$$G_{ij}=\frac{1}{d}\sum_{k=1}^{L}\left[\left(x_{jk}-2p_{k}\right)\left(x_{ik}-2p_{k}\right)\right]{\left[2p_{k}\left(1-p_{k}\right)\right]}^{2\alpha }$$ (4)

where $G_{ij}$ is the genomic relationship between the $i^{th}$ and $j^{th}$ individuals and L is the total number of SNPs, $p_{k}$ is the allele frequency of the *k* ^*th*^ SNP, $x_{jk}$ is the SNP genotype coefficient of the *j* ^*th*^ individual at the *k* ^*th*^ SNP, and d is the expected diagonals computed as $d=L∙E\left[{\left(x_{ik}-2p_{k}\right)}^{2}{\left[2p_{k}\left(1-p_{k}\right)\right]}^{2\alpha }\right]$ . Eq. 4 is implemented in LDAK software (Speed et al., 2012).

Note that Eq. 4 with α = −0.5 is equivalent to the genomic relationship estimation implemented in PLINK, GCTA, and option 2 in BLUPf90 (VanRaden, 2008; Yang et al., 2010; Misztal et al., 2018), and Eq. 4 with α = 0 is equivalent to option 1 in BLUPf90 (VanRaden, 2008; Misztal et al., 2018).

In the HBLUP analysis, we will vary α from −1.5 to 1.5 to find an optimal α value that can improve the phenotypic prediction accuracy and compare the performance with the conventional HBLUP (with α = −0.5 or 0). It is noted that α value is used to estimate GRM using the restricted maximum likelihood (REML) method.

#### 2.3.3 H-matrix (HRM) best linear unbiased prediction

In the HBLUP analysis, GRM (**G**) is computed based on genotypic information, and NRM (**A**) is estimated using the pedigree information of the population. Following Legarra et al., 2009, given estimated **G** and **A** (from Eqs 3, 4), the **H** matrix can be derived as

$$H=\left[\begin{array}{cc} A_{11}+A_{12}A_{22}^{-1}\left(G-A_{22}\right)A_{22}^{-1}A_{21} & A_{12}A_{22}^{-1}\;G\\ GA_{22}^{-1}A_{21} & G \end{array}\right]$$ (5)

In the HBLUP analysis, the simulated data were divided into two groups; one group included the individuals in the first three generations, and the other group included individuals in the last two generations in the current population (101–105th generations). We used the genotypic information of the last two generations and the full pedigree information across the five generations to estimate the **H** matrix. In cattle data, animals available for phenotypes and genotypes were considered (see Table 2) to estimate GRM, and then the HRM was estimated using a combination of NRM estimated based on whole pedigree (84,020 individuals) and GRM.

#### 2.3.4 Blending

GRM is typically a non-positive definite matrix. In the process of HBLUP, it is usually required to modify GRM to be positive definite so that it can be inverted without any numerical problem (VanRaden, 2008). This modification method is called “*blending”* which shrinks the genomic relationships toward the pedigree relationships, using an arbitrary weight, θ , typically ranging from 0.5 to 0.99 (VanRaden, 2008; Meyer et al., 2018; McWhorter et al., 2022). The blended GRM can be written as

$$G_{blended}=\theta G+\left(1-\theta \right)A_{22}\forall 0\leq \theta \leq 1$$ (6)

#### 2.3.5 Tuning

The tuning process adjusts GRM, accounting for the allele frequencies in the base population, using the information from NRM that includes the information of founders in the base population through the pedigree (Legarra et al., 2009; Misztal et al., 2009; Chen et al., 2011; Vitezica et al., 2011; Hsu et al., 2017). The tuned GRM ( $G_{tuned}$ ) is computed as

$$G_{tuned}=\beta G_{blended}+\omega J$$ (7)

where **J** is a matrix with the same size as GRM, all elements are equal to one, and ω and β are tuning parameters that can be used to adjust GRM, accounting for base allele frequencies. In this study, we use the two most frequently used methods to obtain the tuning parameters, ω and β . Following Chen et al., 2011, the first method (referred to as tune = 1) computes ω,and β as

$$\omega =\frac{\left(I^{\prime }A_{22}I-I^{\prime }GI\right)}{n_{2}^{2}}\beta =\frac{\frac{\left[\sum_{i=1}^{n}A_{22_{i,i}}-I^{\prime }A_{22}I\right]}{n_{2}}}{\frac{\left[\sum_{i=1}^{n}G_{i,i}-I^{\prime }GI\right]}{n_{2}}}$$ (8)

where **I** is an array with the size of n×1 and all values equal to one. Following Vitezica et al., 2011, the second method (referred to as tune = 2) can be written as

$$\omega =\frac{\left(I^{\prime }A_{22}I-I^{\prime }GI\right)}{n_{2}^{2}}\beta =1$$ (9)

Please note that Eqs 8, 9 have been implemented in BLUPf90 (Misztal et al., 2018) as the second and fourth tuning options (i.e., TunedG = 2 or 4).

### 2.4 Linear mixed model

In the analyses, we used a linear mixed model that can be written as

y=Xb+Zg+e (10)

where y denotes a vector of phenotypic value, b is a vector of the (environmental) fixed effects, g is a vector of random additive genetic effect that is distributed based on $N\left(0,H\sigma_{g}^{2}\right)$ , where H can be derived from Eq. 5 and $\sigma_{g}^{2}$ denotes the genetic variance. Both X and Z are the incidence matrixes. Finally, the residual effect vector is shown by e distributed as $N\left(0,I\sigma_{e}^{2}\right)$ where I is an identity matrix and $\sigma_{e}^{2}$ is the residual variance.

We employed the restricted maximum likelihood (REML) method, fitting the H matrix, to estimate genetic variance and heritability, which is referred to as HREML in this study. The Akaike Information Criterion (AIC) was used to assess the goodness of fitness of the model as $=2P-2\times \ln\left(L\right)$ , where $\ln\left(L\right)$ is the log likelihood from HREML, and P is the number of parameters. Given the estimated variances and heritability from HREML, HBLUP was used to obtain individual genetic values. We used MTG2.22 (Lee and Van der Werf, 2016; Lee et al., 2017) genomic analysis software to perform the HREML and HBLUP methods.

### 2.5 Grid search to find optimal hyper-parameters

One of the well-known methods to find the best configuration of hyper-parameters is the grid search (LaValle et al., 2014). In the grid search, all possible combinations of hyper-parameters are considered to evaluate the performance of prediction models. We considered two tuning methods and without tuning (Tune = 0, 1, and 2). The blending step size in the grid search is 0.1 from 0 to 1 and 0.02 from 0.9 to 1.0. Meanwhile, the step size for α is 0.1 from −1 and 1.

### 2.6 Key performance metrics

This study uses critical performance metrics to evaluate the accuracy and effectiveness of the prediction and estimation methods. The specific performance metrics will depend on the specific research question, the data type, and the prediction method’s goals. Using multiple performance metrics to provide a more comprehensive assessment of the model’s performance is common.

#### 2.6.1 Root Mean squared error (RMSE)

In genomic analysis studies, we often use a performance metric called RMSE to see how good a model is at making predictions. It's like a measuring stick to compare the model’s guesses with the real answers. We calculate RMSE by taking the differences between the model’s predictions and the actual values, squaring them, and then finding the average of those squared differences. Finally, we take the square root of that average. RMSE is useful because it's simple to understand and tells us how far off the model’s predictions are from the real answers, on average. The smaller the RMSE is related the better the model is at making accurate predictions, which means the guesses are closer to the real answers.

#### 2.6.2 R-value

In the study of genes and their effects on physical traits, scientists often use a tool called the Pearson correlation coefficient (R-value). This helps them figure out if there’s a connection between the two things they’re studying. If the coefficient is high and positive, that means when one thing goes up, the other thing tends to go up too. If it’s high and negative, that means when one thing goes up, the other thing tends to go down.

#### 2.6.3 Akaike information criteria (AIC)

In genomic analysis studies, researchers use statistical models to understand the relationship between genes and traits. The Akaike Information Criteria (AIC) is a metric that compares different models and determines the best one. It was developed by Hirotugu Akaike in 1974 (Akaike, 1974) and is based on the principle of maximum likelihood, which aims to estimate the parameters of a statistical model that is most likely to have produced the observed data. The AIC value represents the amount of information the model loses when it approximates the true underlying process. A lower AIC value indicates a better model fit and a higher likelihood of accurately predicting new data. AIC is a valuable metric for model selection because it takes into account both the goodness of fit and the complexity of the model. It penalizes models with more parameters, which can help prevent overfitting and improve the generalisability of the model to new data. Furthermore, AIC can be used in a wide range of statistical models, including linear regression, generalized linear models, and mixed effects models. It plays a crucial role in model selection, allowing us to choose the model that best fits the data while avoiding overfitting and ensuring that the model is generalizable to new data.

## 3 Results

### 3.1 Simulated data

**FIGURE 2:**
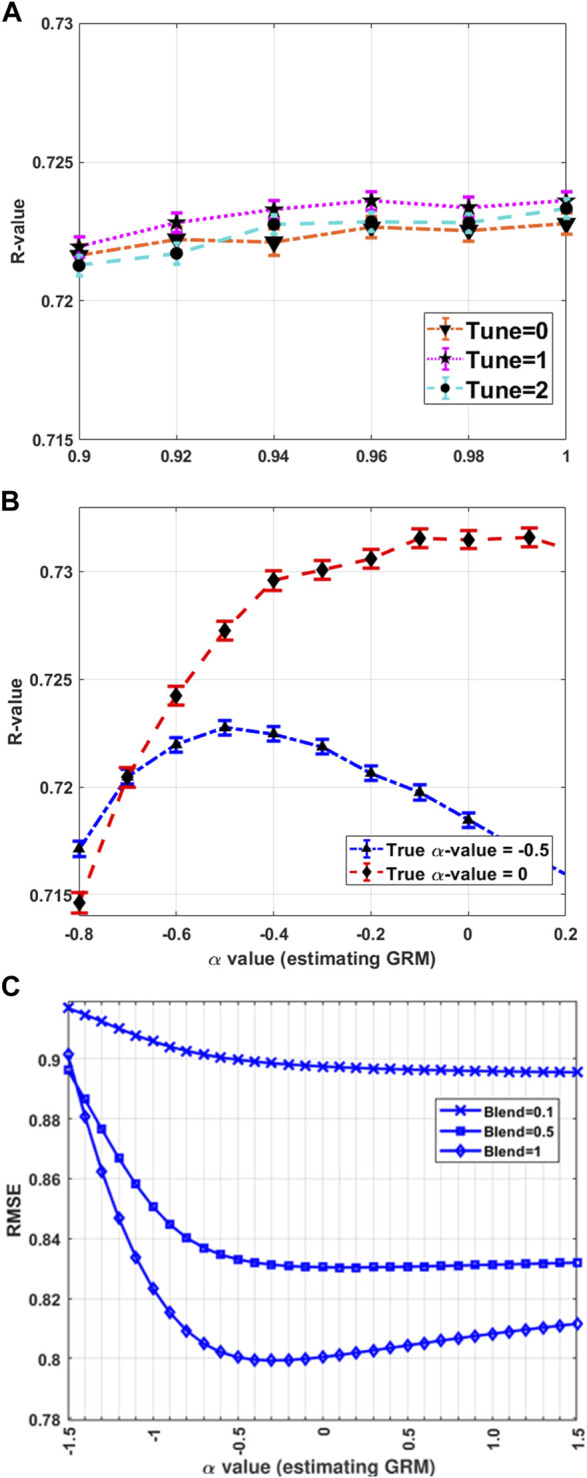
HBLUP accuracy and hyper-parameters. **(A)** The HBLUP accuracy (R-value) improves when using tune = 1 (Eq. 8) or tune = 2 (Eq. 9). However, blending ( θ < 1) would not increase the accuracy for this simulated dataset. **(B)** Optimal α values can increase the accuracy, and also **(C)** can decrease the RMSE indicating that the choice of α is important in HBLUP. We simulated genotypes and phenotypes in 3,000 replications in which simulation parameters of $h^{2}=$ 0.8, $N_{e}=100$ for 100 historical generations and a half-sib design (50 male individuals, 500 female individuals) were used. The true α values used in the phenotypic simulation were −0.5 or 0. The error bars are 95% CI over the 3,000 replications.

Figure 2A shows that the tuning process can improve the phenotypic prediction accuracy (referred to as R-value) when using the simulated data, which is a Pearson correlation coefficient between the observed and predicted phenotypes in the target dataset, confirming previous studies. However, it should be noted that the improvement in prediction accuracy between Blend = 0.9 and Blend = 1 is only 0.003, which may be considered relatively small. The tuning process with the first option (tune = 1; Eq. 8) appears to better perform than the second option (tune = 2; Eq. 9) for this simulated data. However, this shows that tuning GRM before blending had a negligible impact on genomic predictions (McWhorter et al., 2022). Furthermore, blending ( θ <1) does not significantly improve the HBLUP accuracy for this simulated data (Figure 2A; Supplementary Figure S2). Figure 2B represents the impact of α value on the HBLUP’s performance, showing that the prediction accuracy increases when α value used in estimating GRM is close to the true α value used in the phenotypic simulation. When varying simulation scenarios (e.g., a small or large effective population size with full-sib designs), a similar result is observed: the phenotypic prediction accuracy improves when applying the tunning process or when using optimal α (Supplementary Figures S3–S6). In addition, Figure 2C shows the importance of α value in decreasing the root mean square error (RMSE) prediction of the HBLUP, and Blend = 1 proposes less RMSE compared with Blend = 0.1 and Blend = 0.5.

**FIGURE 3:**
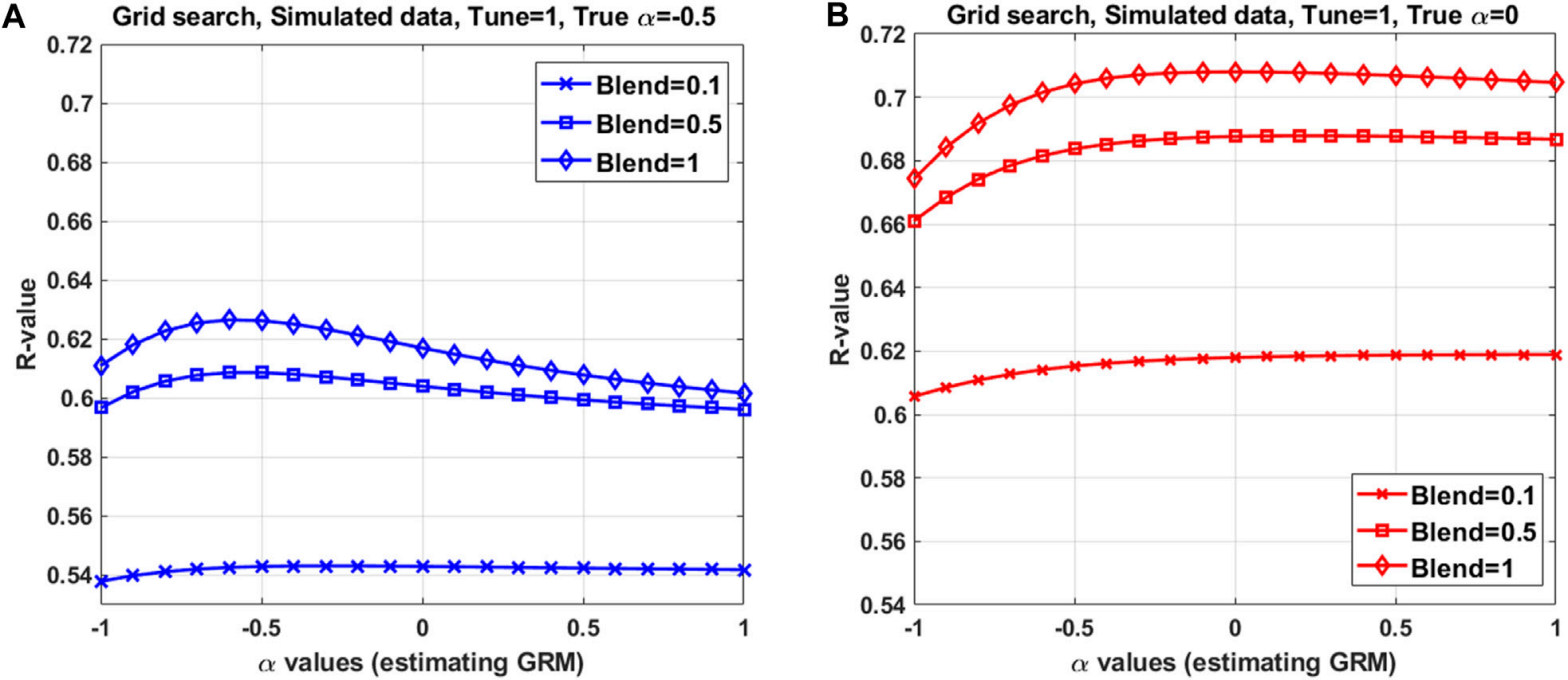
HBLUP accuracy averaged over 5-fold cross validation in a grid search with various configurations of the hyper-parameters, using a single simulation dataset. The best configuration found in the grid search consists of **(A)** and tune = 1, blend = 0.9, and *α* = −0.5 when using *α* = −0.5 in the simulation, and **(B)** tune = 1, blend = 1 and *α* = 0 (in estimating GRM) when using *α* = 0 in the simulation. The population parameters used in the simulation are *h^2^* = 0.8, *N_e_* = 100 for 100 historical generations, N_SNPs_ = 9000, chromosome number = 30 and *α* = 0 or −0.5. Mimicking livestock population, a half-sib design (50 sires, 10 dams per sire and 2 offspring per dam) was applied to the last 5 generations. Full pedigree across the 5 generations were used in HBLUP. Among 2000 offspring in the last 2 generations, 5 subsets each with a random 400 individuals were used as target datasets in the 5-fold cross validation. To predict for each target dataset, the remaining 5150 (across the 5 generations) were used as the discovery dataset.

Mimicking a real dataset in which multiple replicates are not possible, we used a single simulation data to assess the HBLUP accuracy, varying hyper-parameters (Figure 3). All possible configurations of tuning, blending, and α values were evaluated using the grid search method where the prediction accuracy was measured using 5-fold cross-validation (see Methods and Supplementary Figures S7, S8). Figure 3 shows the HBLUP accuracy averaged over 5-fold cross-validation when varying hyper-parameters. The highest phenotypic prediction accuracy was achieved with tune = 1, blend = 1, and α = 0 when using the true α = 0, and with tune = 1, blend = 0.9, and α = −0.5 when using the true α = −0.5 in the simulations (See Figure 3 and Supplementary Figures S9, S10 for average RMSE). This shows that the optimal α values found in the grid search are approximately in agreement with the true simulated values.

### 3.2 Cattle data

**FIGURE 4:**
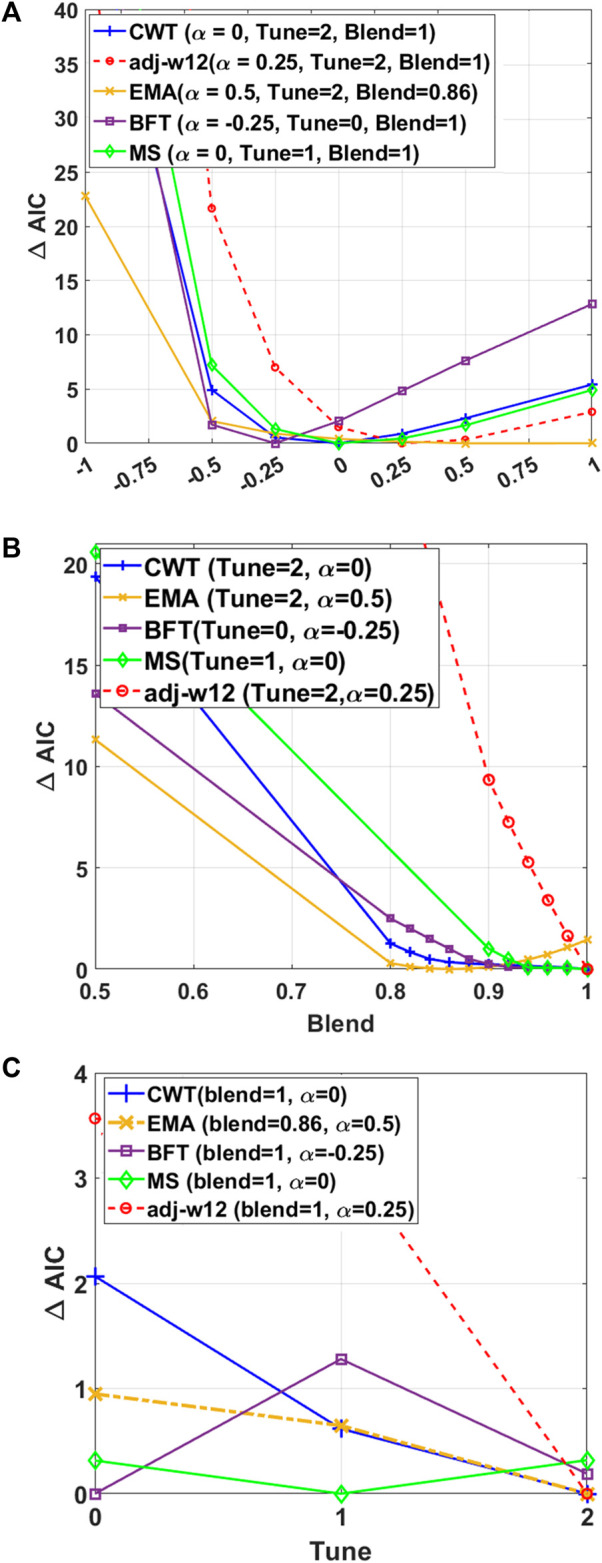
HREML estimation accuracy depends on α estimated in the genotyped samples and HRM. **(A)** Evaluating the impact of α values on the ∆AIC for five different traits of the Hanwoo cattle dataset using HREML in a univariate linear mixed model with different tuning methods and blending coefficients. The Akaike Information Criterion (AIC) was used to show the goodness of fitness of the model as $AIC=2P-2\times \ln\left(L\right)$ , where $2\times \ln\left(L\right)$ is the HREML log likelihood, and P is the number of parameters. $∆AIC=AIC-{AIC}_{optimal}$ , where AIC is obtained with the corresponding α value at the x-axis and ${AIC}_{optimal}$ is the AIC for the optimal α . It is observed that optimal α varies across traits. Whole individuals with available phenotypes were applied in estimating the heritability based on Table 2. **(B)** A performance comparison between two different blending coefficients (0.5–1) in order to estimate the HRM using HREML with optimal tuning method and optimal α value. **(C)** The performance of tune = 1 (Eq. 8) compared with the tune = 2 (Eq. 9), without considering tuning in estimating the HRM with the applied optimal blending and α values.

We used pedigree, genotype, and phenotype data of Korean native cattle (Hanwoo), which is a unique and important breed in the beef industry (Kim et al., 2017; Srivastava et al., 2021), to assess the HBLUP accuracy with various hyper-parameters including α . We first estimated optimal hyper-parameters that provided the lowest Akaike information criteria ( AIC ) value based on the residual maximum log-likelihood for each trait, using HREML (Figure 4). We observed that ∆AIC was not uniformly distributed across different α values, and optimal α values were largely different across five carcass traits (Figure 4A). On the other hand, a blending parameter θ = 1 provided the lowest ∆AIC values for all traits except for EMA ( θ = 0.86), indicating that a blended GRM with θ < 1 did not increase the goodness of fit when using HREML in general (Figure 4B). Finally, Figure 4C shows that tune = 2 could achieve better goodness of fit, compared with tune = 1 or tune = 0 (i.e., without tuning), in most cases. For BFT and MS traits, tune = 1 and 0 provided the lowest AIC (Figure 4C) although the AIC was not significantly lower than tune = 2 (difference in AIC less than 1). The best-performed hyper-parameters for five traits can be seen in Supplementary Table S1.

**FIGURE 5:**
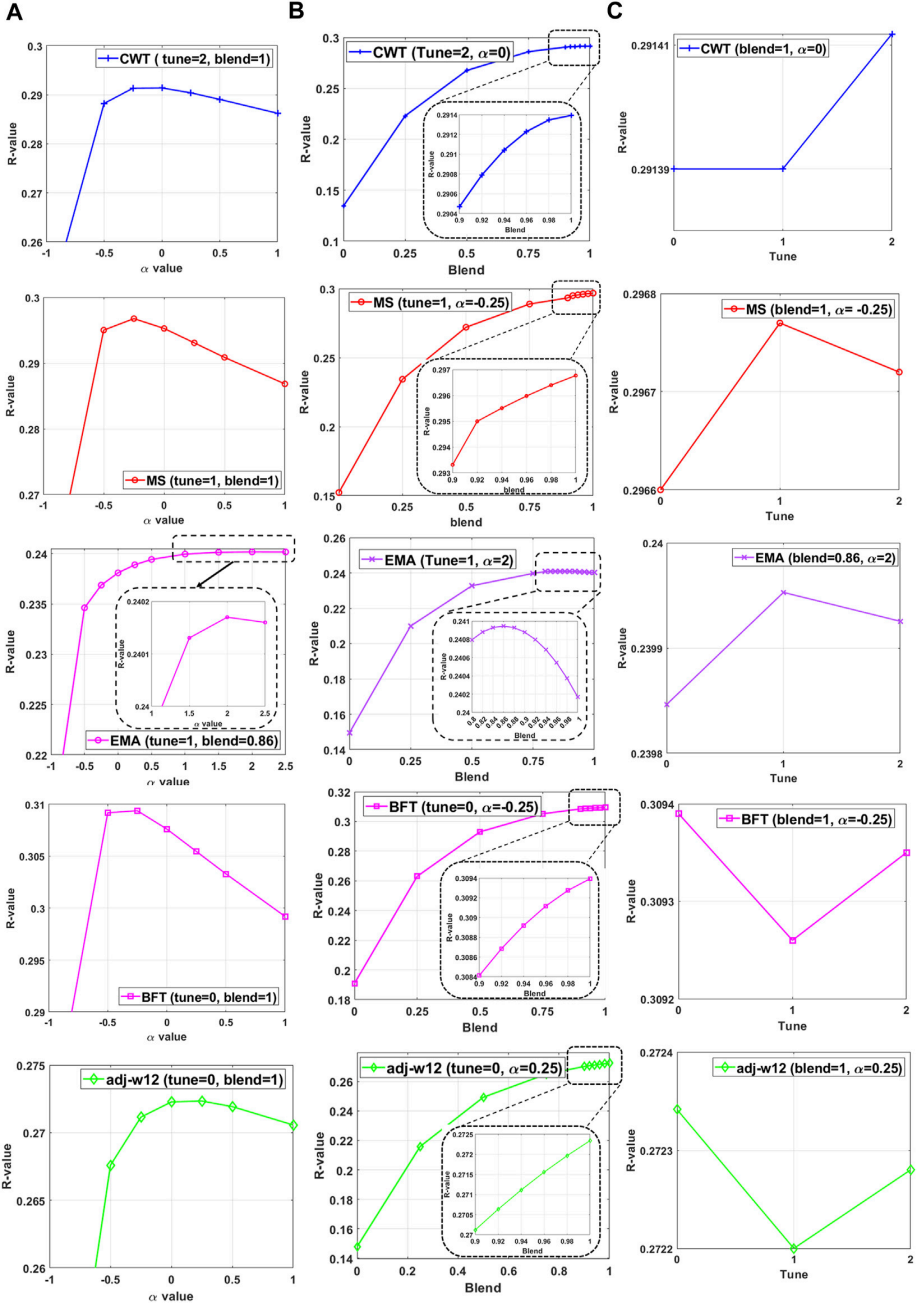
The performance of HBLUP when **(A)** varying α , **(B)** blending, and **(C)** tuning hyper-parameters for five carcass traits. The five carcass traits include carcass weight (cwt), eye muscle area (ema), adjusted 12 months weight (adj-w12), marbling score (ms), and back fat thickness (bft). There are a total of 84,020 animals in the Hanwoo cattle pedigree, of which 9,072 animals have both phenotypic and genotypic records that are randomly divided into five validation groups (Table 2). Each set of the five groups is selected as the target samples, and all the phenotyped animals except the target samples were used as the discovery dataset. This 5-fold cross-validation was used to validate the performance of HBLUP.

We also used a grid search to assess the performance of all hyper-parameters (Figure 5) in which HBLUP accuracies of all possible configurations of tuning, blending, and α values were evaluated in 5-fold cross-validation. Figure 5 shows the HBLUP accuracy averaged over 5-fold cross-validation when varying α , tuning, and blending values for five carcass traits. In Figure 5A, we observed that the accuracy of HBLUP could be considerably increased or decreased, depending on the choice of α values. In contrast, Figure 5B shows that the highest HBLUP accuracy was achieved with a blending parameter θ = 1 for all traits except EMA ( θ = 0.86), indicating that blended GRM would not improve the HBLUP accuracy in most cases. Finally, Figure 5C indicates that the tuning process would not substantially improve the HBLUP accuracy for all carcass traits in Hanwoo cattle data. In addition, the 2-D landscape of grid search results for HREML estimation accuracy depending on α estimated in the genotyped samples and making HRM can be seen in Supplementary Figure S11. The best configuration of the hyper-parameters for each trait is shown in Supplementary Table S1.

## 4 Discussion

HBLUP or ssGBLUP has been widely used in livestock breeding programs (De Los Campos et al., 2009; VanRaden et al., 2009). The HBLUP method (e.g., BLUPf90) requires hyper-parameters to integrate the information of genomic and pedigree relationship matrices, which should be optimised to increase the accuracy of genomic prediction (Legarra et al., 2009; Chen et al., 2011; Vitezica et al., 2011; Meyer et al., 2018). In this study, we evaluated the performance of HBLUP with various hyper-parameters such as blending, tuning, and scale factor, using simulated and real Hanwoo cattle datasets.

In our simulation scenario, we employed random mating and random selection instead of artificial selection based on phenotypes or estimated breeding values because the purpose of this simulation study is to demonstrate how the novel hyper-parameter, alpha, works in a simplified simulation setting. Nevertheless, we have applied our approach to real cattle data that have been subjected to artificial selection. By doing so, we believe that we have verified the performance of the hyper-parameter in a realistic setting. In both simulation and real data, allele frequencies can be altered significantly due to genetic drift and selection (Falconer and Mackay, 1996; Hartl and Clark, 1997; Lynch and Walsh, 1998).

The scale factor, α , can determine the relationship between allele frequency and per-allele effect size. In the simulation, HBLUP accuracy can be the highest when using GRM scaled by the true α value used in the phenotypic simulation, indicating that the choice of α value is important although this has never been considered as a hyper-parameter in HBLUP. In fact, the performance of HBLUP is shown to vary across the carcass traits in the cattle data used in this study, confirming previous studies that reported that optimal α values vary, depending on traits and populations (Speed et al., 2012; Speed et al., 2017; Momin et al., 2023). Importantly, using less optimal α values may decrease HBLUP accuracy significantly, which should be carefully checked before conducting genetic evaluations, emphasising that the scale factor is not less important, compared to other hyper-parameters such as blending and tuning.

In both simulated and cattle data, blending ( θ<1 ) would not really improve the phenotypic prediction accuracy except that of the cattle traits, EMA; the blending of $\theta_{optimal}=0.86$ could increase the accuracy. The accuracy would increase more when GRM was blended with higher weights, which is clearly shown in Supplementary Figure S2. This is not totally unexpected because richer information can come from GRM (e.g., Mendelian sampling variance within sibs), and blended GRM may lose some of such information. When the mixed model equation is used for HREML or HBLUP (Henderson, 1953; Misztal et al., 2018), a non-positive definite GRM may cause a numerical problem, for which the blending process is essential. This may be one of the reasons blending has been an important hyper-parameter in HBLUP. However, the direct Average Information algorithm can use a non-positive definite GRM without blending ( θ=1 ) and there is a method that can provide a positive definite GRM (Momin et al., 2023). In any case, we recommend optimising the blending hyper-parameter as the optimal blending can vary, depending on data, in which θ=1 should also be explicitly evaluated. A non-positive definite GRM means the matrix has one or more negative eigenvalues, which can cause problems in certain computations. For example, when using the GRM in a linear mixed model for genomic prediction, the non-positive definiteness can lead to negative variance estimates, which are not biologically meaningful (Legarra et al., 2009). In addition, non-positive definite matrices can cause numerical instability in various computations, such as matrix inversion and eigenvalue decomposition (Kang et al., 2008). One common cause of a non-positive definite GRM is the presence of genotyping errors, which can lead to negative pairwise genetic distances between individuals. Small sample sizes, genotyping errors, and mean bias from the current GRM method can contribute to this problem (Momin et al., 2023). However, there is a reliable algorithm available to address non-positive definite GRMs, which is the direct average information algorithm (Lee and Van Der Werf, 2006; Yang et al., 2011; Lee and Van Der Werf, 2016).

The tuning process adjusts GRM, accounting for the allele frequencies in the base population, assuming that the founders in the base population are not genotyped but are linked through the pedigree. As expected, the widely used tuning method (tune = 1 (Chen et al., 2011) implemented in BLUPf90 option 2) could improve the prediction accuracy in the simulated data, indicating that the base allele frequencies are correctly accounted for. However, the improvement caused by tune = 1 or 2 was not remarkable in the Hanwoo cattle data. This is probably due to the fact that the pedigree information in the real data is not accurate enough to trace the founders, or the genotypes may capture substantial information about the base allele frequencies.

The grid search benefits include being able to provide reproducible results, being fast to implement, being simple to develop for parallel computing, and being efficient in exploring a low-dimensional hyper-parameter space. Moreover, for the large-scale hyper-parameters search space, there are a large number of other hyper-parameters optimisation methods, such as genetic/evolutionary algorithms, swarm intelligence methods, stochastic/random search techniques, and co-evolutionary algorithms (Dudzik et al., 2021). These methods are able to provide robust performance in exploring the multi-modal search space (Kuyu and Vatansever, 2021).

In conclusion, existing hyper-parameters such as blending and tuning in HBLUP are important in general, and their optimal values or options should be properly sought to achieve a reliable genetic evaluation. Depending on the data, optimal values can vary, and unnecessary or over-parametrised blending or tuning can produce adverse effects on the prediction accuracy. The scale factor, a novel hyper-parameter to be introduced in HBLUP, should be explicitly optimised to increase the prediction accuracy, given that the impact of the scale factor is competitive with other hyper-parameters, blending and tuning. We suggest including the scale factor, α , in HBLUP as a hyper-parameter.

## Data Availability

The data analyzed in this study is subject to the following licenses/restrictions: The SNP genotypic data and phenotypic data of Korean Hanwoo cattle used in this study are deposited and available at the digital repository of NIAS, South Korea (https://www.nias.go.kr/). Moreover, the simulated data generated and used in this study is available by https://github.com/a1708192/HBLUP_gridsearch.git. Requests to access these datasets should be directed to SoL, lhyungm@korea.kr (lhyungm@gmail.com https://www.nias.go.kr/.
